# Case Report: Hypercalcemic small-cell carcinoma of the ovary during pregnancy: diagnostic and therapeutic challenges

**DOI:** 10.3389/fonc.2025.1648580

**Published:** 2025-08-06

**Authors:** Marta Tripepi, Ana G. da Costa, Diogo Albergaria, Andreia Costa, Ana Catarino, Ana Luisa Duarte, Carla Bartosch, Veronica Schuler, Joana Oliveira, Jorge Lima, João Casanova

**Affiliations:** ^1^ Department of Women and Children’s Health, Clinic of Gynecology and Obstetrics, University of Padua, Padua, Italy; ^2^ Gynecologic Oncology Unit, Obstetrics and Gynecology Service, Department of Surgery, Hospital da Luz Lisboa, Lisbon, Portugal; ^3^ General Surgery Unit, Department of Surgery, Hospital da Luz Lisboa, Lisbon, Portugal; ^4^ Department of Oncology, Centro Hospitalar Universitário de São João, Porto, Portugal; ^5^ Gynecologic Oncology Unit, Department of Pathology, Hospital da Luz Lisboa, Lisbon, Portugal; ^6^ Department of Radiology, Centro Hospitalar Universitário de São João, Porto, Portugal; ^7^ Department of Pathology, Portuguese Institute of Oncology Porto, Porto, Portugal; ^8^ Department of Anesthesiology, Hospital da Luz Lisboa, Lisbon, Portugal; ^9^ Obstetrics and Gynecology Service, Department of Surgery, Hospital da Luz Lisboa, Lisbon, Portugal; ^10^ Comprehensive Health Research Centre (CHRC), NOVA Medical School|Faculdade de Ciências Médicas (NMS|FCM), Universidade Nova De Lisboa, Lisbon, Portugal

**Keywords:** small cell ovarian cancer hypercalcemic type, ovarian neoplasms, cancer and pregnancy, cytoreductive surgery, rare ovarian neoplasms

## Abstract

Small-cell carcinoma of the ovary, hypercalcemic type (SCCOHT), is a rare, highly aggressive malignancy that predominantly affects young women. We report a 32-year-old pregnant woman diagnosed with SCCOHT during the first trimester of pregnancy. At 24 weeks, imaging revealed extensive peritoneal carcinomatosis. Following multidisciplinary evaluation, neoadjuvant chemotherapy was initiated, but the disease progressed. At 34 weeks, the patient underwent cesarean delivery followed by cytoreductive surgery. Despite achieving an initial complete resection, the disease recurred rapidly. The patient died shortly after completing adjuvant chemotherapy and initiating immunotherapy. This case highlights the diagnostic and therapeutic challenges of managing SCCOHT during pregnancy and the complex balance of maternal and fetal outcomes. Early diagnosis, coordinated multidisciplinary care, and timely intervention are critical, although the prognosis remains poor despite aggressive multimodal treatment.

## 1 Introduction

Small-cell carcinoma of the ovary, hypercalcemic type (SCCOHT), was first described by Dickersin et al. in 1982 (1). It is an exceptionally rare and aggressive malignancy, representing less than 0.01% of all ovarian cancers (2). The peak incidence is approximately 24 years of age, although rare cases have been reported in children (1).

SCCOHT often presents with non-specific symptoms such as abdominal pain, bloating, nausea, vomiting, and fatigue. Approximately two-thirds of patients exhibit hypercalcemia, which can lead to serious complications such as pancreatitis and altered mental status (3).

In 2014, inactivating mutations of the *SMARCA4* gene were identified as the genetic hallmark of SCCOHT (4–6). This discovery has significantly improved diagnostic accuracy, enabling distinction from other poorly differentiated ovarian neoplasms through immunohistochemical and molecular profiling. Despite advances in molecular understanding, the prognosis for SCCOHT remains poor, with an overall 5-year survival rate of less than 35% in International Federation of Gynecology and Obstetrics (FIGO) stage IA and under 10% in advanced-stage disease (3, 7). Standard treatment typically incorporates multimodal therapy, like surgery and chemotherapy. In rare cases, radiotherapy is utilized. Given its rarity, non-specific clinical presentation, and aggressive behavior, SCCOHT continues to pose significant diagnostic and therapeutic challenges.

## 2 Case presentation

**Figure 1:**
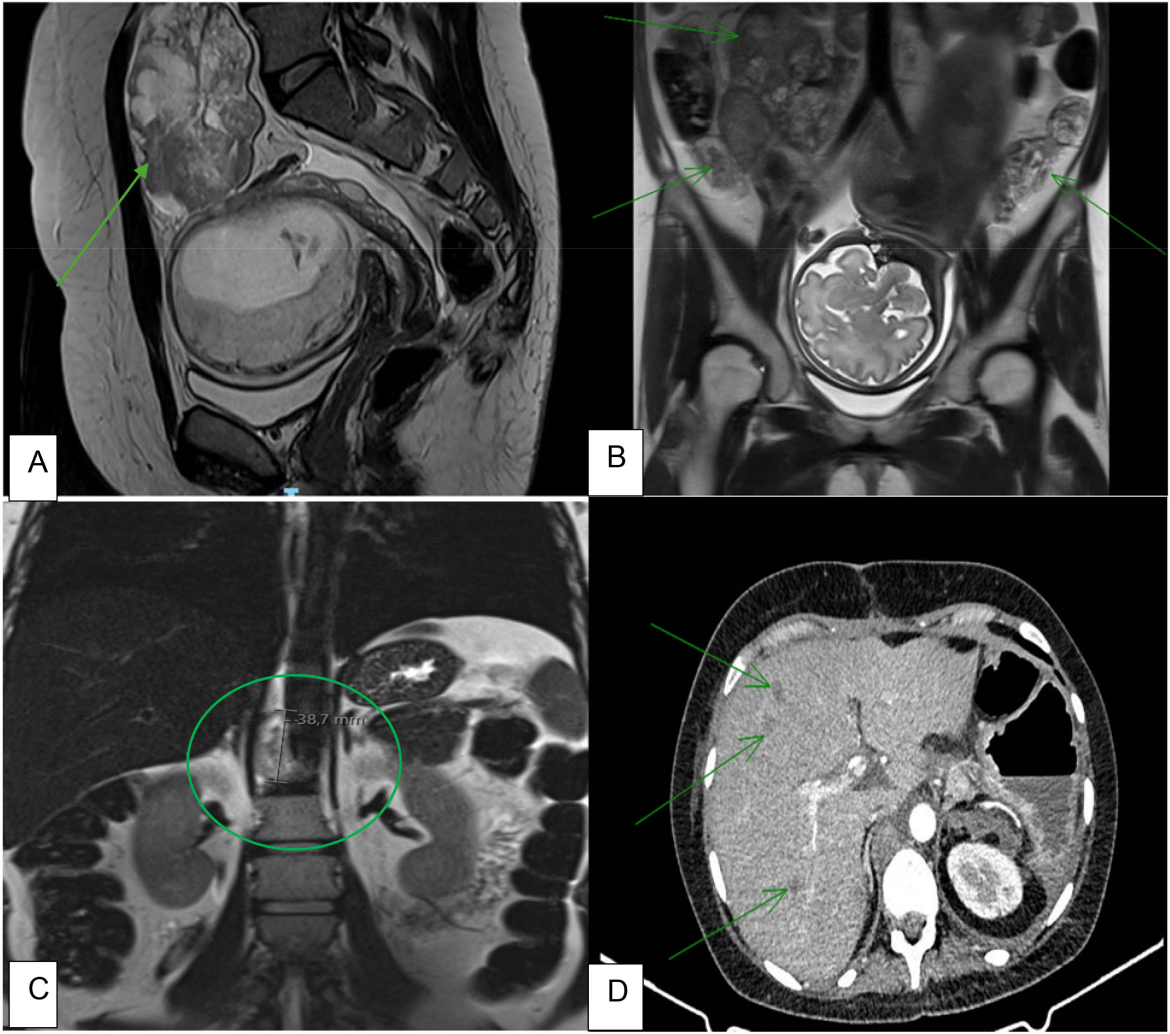
Imaging highlighting different stages of disease. **(A)** MRI performed during the first trimester of pregnancy showing a viable pregnancy and an ovarian tumor classified as ORADS-4. **(B)** MRI performed after the second cycle of carboplatin and paclitaxel, highlighting peritoneal carcinomatosis (arrow). **(C)** MRI performed after the second cycle of carboplatin and paclitaxel showing a retrocrural adenopathy, compatible with disease progression. **(D)** CT scan performed 3 weeks after surgery, showing evidence of disease progression and liver metastases (arrow).

A 32-year-old pregnant woman underwent a first-trimester pregnancy ultrasound (US) at an outside hospital. The US revealed an 8-cm solid right ovarian mass. She subsequently underwent a pelvic magnetic resonance imaging (MRI), without gadolinium-based contrast, which classified the tumor as intermediate-risk for malignancy (ORADS 4) as per the Ovarian-Adnexal Reporting Data System (**Figure 1A**). CA125 was 36.5 kU/L. A right oophorectomy was performed at a different medical center, and pathology revealed a ruptured tumor, compatible with small-cell carcinoma of the ovary hypercalcemic type. The immunohistochemistry showed the following: CAM5.2, AE1/AE3, CK7, beta-catenin, calretinin, synaptophysin, smooth muscle actin (SMA): positive multifocal; EMA, inhibin alpha: positive focal; vimentin: positive, more intense in large cells; WT1 positive diffusely in small cells; multifocal in large cells; CD99: positive, membrane multifocal; chromogranin, S100, PAX-8, myogenin and MyoD1, RE and R+: negative; p53: wild type (positive 30%–40%); p16: mosaic expression; SMARCA4: loss of expression; MLH1, PMS2, MSH2, MSH6: without expression alterations.

Of note, her past medical history was notable for two prior conizations for adenocarcinoma *in situ*.

At 24 weeks of gestation, 2 months after the first surgery, the patient presented to our clinic for a second opinion. She had undergone another pelvic and abdominal MRI (without gadolinium-based contrast) showing retroperitoneal lymphadenopathies and peritoneal carcinomatosis. After a multi-disciplinary team (MDT) discussion that considered the patient’s desire to proceed with the pregnancy, she started chemotherapy with carboplatin and paclitaxel.

After two cycles of chemotherapy, her pelvic pain worsened, and disease progression was notable on repeat abdominal and pelvic MRI (without gadolinium-based contrast) (**Figures 1B, C**). The MDT recommended a scheduled cesarean delivery at 34 weeks with concurrent cytoreductive surgery.

**Figure 2:**
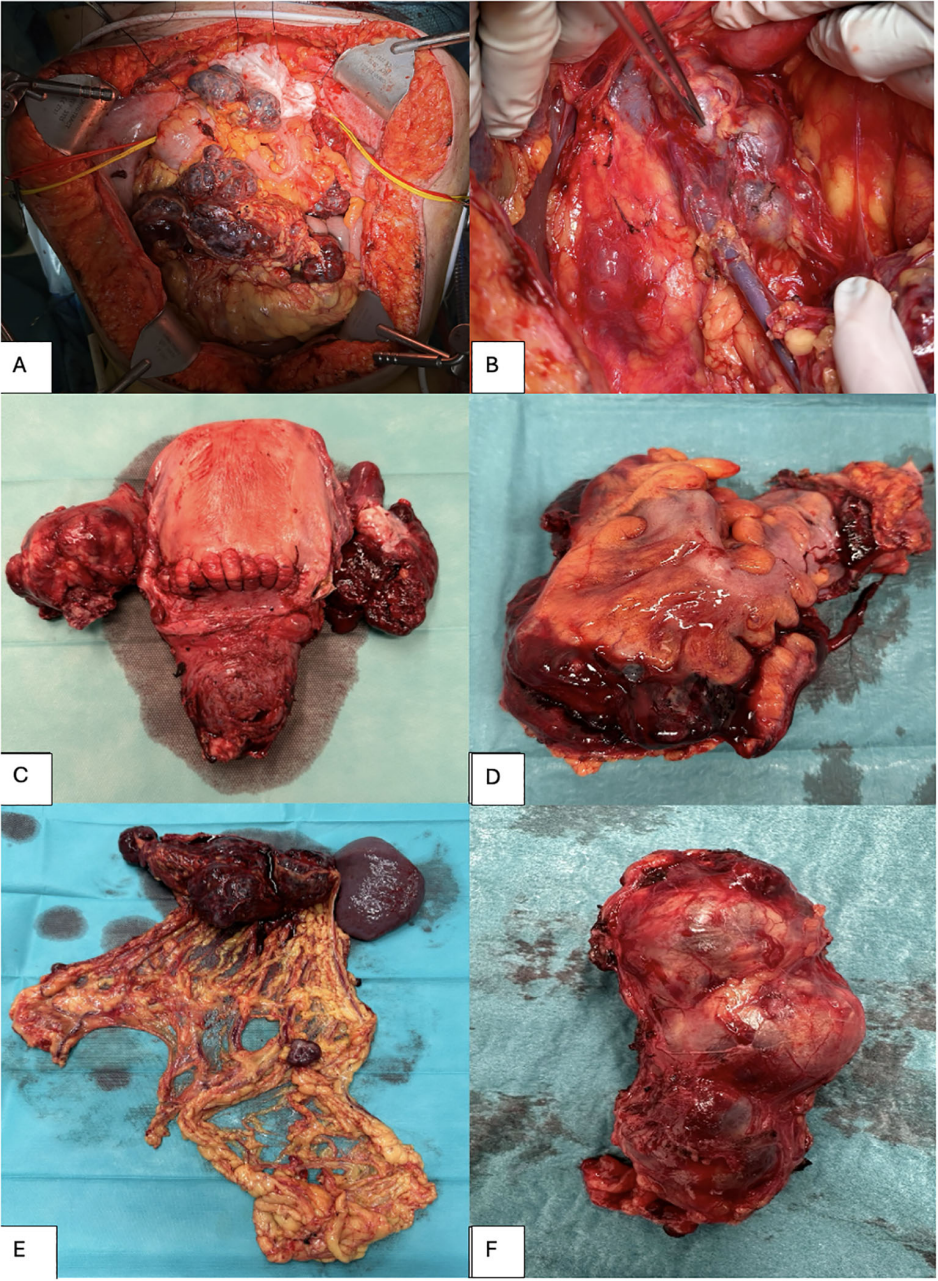
Surgical specimens removed during cytoreductive surgery. **(A)** Abdominal cavity. **(B)** Pathologic lymph nodes, approximately 8 cm in size, adhering to the inferior vena cava (VCI) and the aorta. **(C)** Hysterectomy and bilateral salpingo-oophorectomy. **(D)** Rectosigmoid colon. **(E)** Infragastric omentectomy and splenectomy. **(F)** Resected lymph node package adhering to the inferior vena cava (VCI) and the aorta.

The patient underwent a midline laparotomy, and a cesarean section was performed first. Afterward, the incision was extended to the xiphoid, and the peritoneal cavity was thoroughly explored (**Figure 2A**). The surgical team decided to perform a hysterectomy first due to an increased risk of bleeding. The retroperitoneum was opened, and both common and internal iliac arteries were secured with a vessel loop to maintain vascular control in case of acute uterine bleeding. Hysterectomy with bilateral salpingo-oophorectomy (BSO) was performed (**Figure 2C**). The surgery proceeded with upper abdominal cytoreduction, including en bloc splenectomy and infragastric omentectomy (**Figure 2E**). After fully mobilizing the liver and opening the lesser sac, the right crus of the diaphragm was identified and sharply incised. A 3–4-cm nodule adhering to the aorta and spine was resected.

The patient underwent appendectomy, sigmoid resection, and resection of several bulky retroperitoneal lymph nodes (**Figures 2B, D, F**), both pelvic and para-aortic. Additionally, resection of abdominal wall implants was performed, followed by mesh reconstruction. Due to significant blood loss (1,250 mL, intraoperative hemoglobin of 4.6), three units of packed red blood cells were given, and a diverting ileostomy was created. A complete cytoreduction was achieved with no gross residual disease.

The postoperative period was complicated by a left renal vein thrombosis and abdominal wall seroma requiring image-guided drainage. A superficial 2–3-cm wound dehiscence was noted. In total, she received seven units of packed red blood cells.

**Figure 3:**
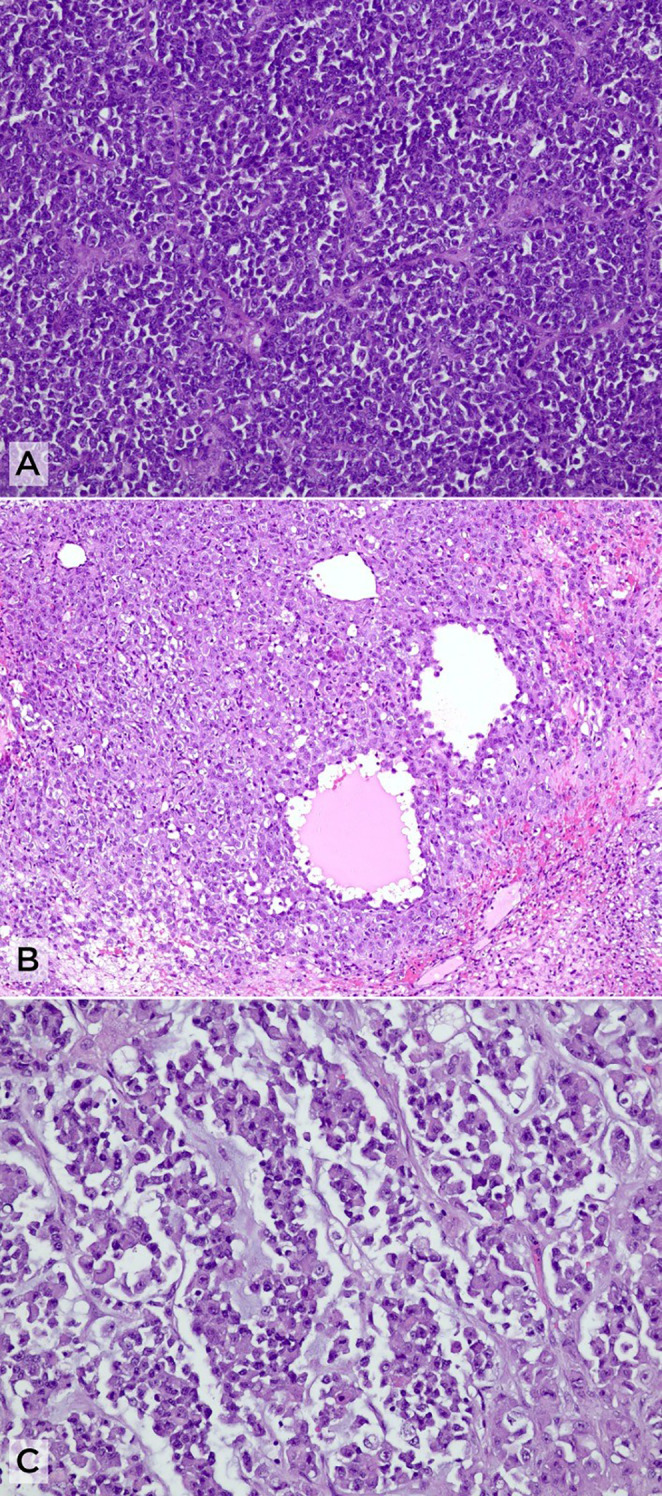
Histopathological features of small-cell carcinoma of the ovary, hypercalcemic type (SCCOHT). **(A)** Solid sheets of small, round-to-oval cells with scant cytoplasm (H&E, ×10). **(B)** Pseudofollicular arrangements surrounded by a population of larger cells with more abundant cytoplasm (H&E, ×20). **(C)** Rhabdoid morphology with eccentric nuclei and abundant eosinophilic cytoplasm in a myxoid background (H&E, ×20).

**Figure 4:**
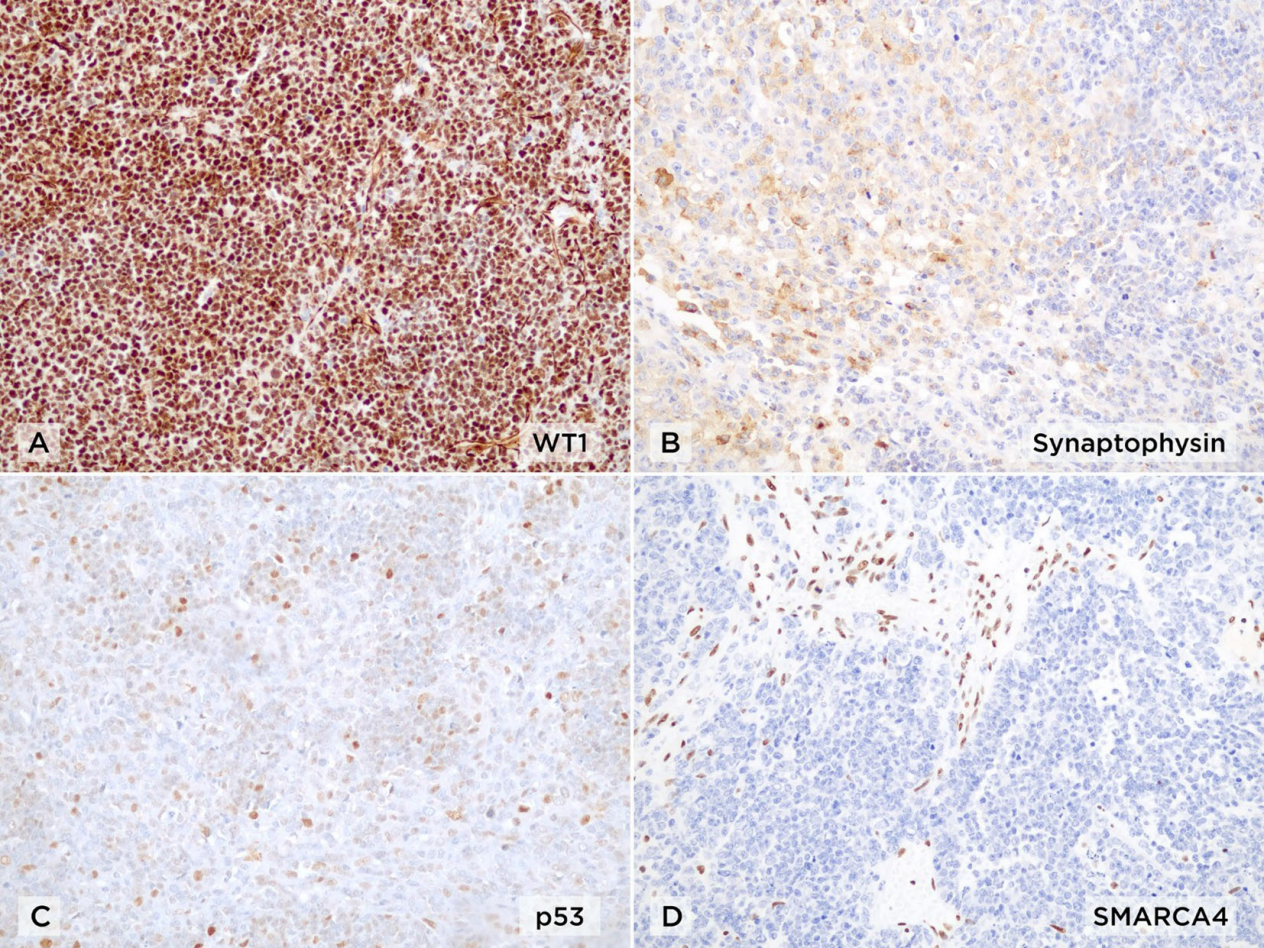
Immunohistochemical profile of small-cell carcinoma of the ovary, hypercalcemic type (SCCOHT) (×20). **(A)** Diffuse nuclear positivity for WT1. **(B)** Multifocal staining for synaptophysin. **(C)** Wild-type pattern of p53 expression. **(D)** Complete loss of nuclear SMARCA4 (BRG1) expression in tumor cells, with retained expression in stromal and endothelial cells as internal positive control.

Three weeks postoperatively, during follow-up, a chest–abdomen–pelvis (CAP) computed tomography (CT) scan demonstrated multiple liver lesions suggestive of metastases and suspicious retrocaval and pelvic lymphadenopathy (**Figure 1D**). She was discharged on postoperative day 21. Final pathology confirmed SCCOHT, FIGO stage IVB (abdominal wall metastasis) (**Figures 3**, **4**). The patient started adjuvant chemotherapy with bleomycin, etoposide, and cisplatin (BEP) at 65 days after surgery and completed six cycles. Two weeks after the last cycle of chemotherapy, she underwent a CAP CT scan that revealed a mixed response, with smaller peritoneal implants and retroperitoneal adenopathies but increased size of the liver metastases. She started nivolumab as maintenance therapy at 20 days after chemotherapy. Two weeks after the first cycle of nivolumab, she was hospitalized for acute renal failure, multi-organ failure, and disease progression. The patient died a few days after being admitted to the hospital.

## 3 Discussion

SCCOHT is an extremely rare and aggressive ovarian malignancy. The 2014 World Health Organization (WHO) classification includes it among miscellaneous ovarian neoplasms (8). The age distribution at diagnosis is remarkably heterogeneous, with reported cases ranging from 7 months to 56 years, yielding a mean age of approximately 23.9 years (3). There are no well-established environmental or lifestyle risk factors for small-cell carcinoma of the ovary (9). Patients typically present with non-specific abdominal pain, bloating, vomiting, and nausea. Hypercalcemia occurs in approximately two-thirds of all cases, often in early disease stages. It can be helpful in diagnosing SCCOHT. Hypercalcemia is also associated with lethargy, polyuria, polydipsia, constipation, confusion, and, in some cases, pancreatitis or altered mental status (2). Preoperative assessment should follow published guidelines for epithelial ovarian cancer, including imaging studies and tumor markers (10). The initial imaging evaluation should include abdominal and transvaginal ultrasound (11). Inexplicably, most reported cases of SCCOHT occur on the right ovary (3). Given the highly aggressive nature of the disease, the use of MRI or CT scan is essential for staging and treatment planning (7). These imaging studies allow for the identification of suspicious lymph nodes, distant organ metastases, ascites, or peritoneal carcinomatosis. Positron emission tomography (PET) scan can also be utilized to evaluate for metastatic spread (7).

Distinguishing SCCOHT from other non-epithelial ovarian tumors using only imaging and tumor markers is difficult. Routinely determining blood biomarkers, such as CA125, carcinoembryonic antigen (CEA), alpha-fetoprotein (AFP), beta-human chorionic gonadotropin (b-HCG), and lactate dehydrogenase (LDH), is recommended, like for epithelial and non-epithelial ovarian cancers (10). Histological examination with immunohistochemical markers remains the gold standard for the diagnosis of SCCOHT (12). Histologically, SCCOHT consists of small, monotonous cells, sometimes with follicle-like spaces. As mentioned earlier, a hallmark of SCCOHT is the biallelic inactivation of the *SMARCA4* gene, resulting in the complete loss of BRG1 expression—the catalytic subunit of the SWI/SNF chromatin-remodeling complex responsible for regulating gene transcription (9, 13). Despite that these mutations are crucial for diagnosing SCCOHT, they may not be universally feasible in the clinical setting. According to the literature, SMARCA4 immunohistochemistry (IHC) is a highly sensitive and specific test for the diagnosis of SCCOHT. Published data have shown that the sensitivity and specificity of SMARCA4 IHC were excellent at 88% and 94%, respectively (14). In most SCCOHT cases, conservative management is generally not recommended. Surgical treatment, even for clinical stage I disease, should include total abdominal hysterectomy and bilateral salpingo-oophorectomy with peritoneal staging and full pelvic and para-aortic lymphadenectomy (15). Lymph node metastases are usually less frequent in these tumors compared to high-grade serous carcinoma; however, as Takeshima et al. explained, the rationale for performing a systematic lymphadenectomy lies in the fact that these tumors are often chemoresistant (16, 17). For advanced-stage disease, cytoreductive surgery is advocated despite the lack of robust data. If complete cytoreduction is not considered feasible as a first option, it is advisable to start neoadjuvant chemotherapy and subsequently evaluate for resectability (18). Because SCCOHT is extremely rare, there are several regimens of adjuvant therapy described in the literature. Due to the highly aggressive nature of the disease, adjuvant chemotherapy is recommended even for patients diagnosed at an early stage (9). A commonly used strategy is to manage SCCOHT similarly to small-cell lung cancer, employing a combination of cisplatin (or carboplatin) and etoposide. This treatment regimen is considered appropriate due to the “small cell” histological features and has been applied in several cases, sometimes with the addition of a third agent such as cyclophosphamide (19). The rationale to utilize other regimens is often extrapolated from protocols for other high-grade ovarian malignancies (platinum plus paclitaxel chemotherapy regimen) (20). Senekjian et al. evaluated the effectiveness of a six-drug chemotherapy regimen. The regimen consisted of vinblastine and cisplatin at induction, followed by cyclophosphamide and bleomycin at 24 hours and doxorubicin and etoposide at 36 hours for a total of six cycles (21). Another regimen was proposed by Pautier et al. and included cisplatin and Adriamycin on day 1 and VePesid and cyclophosphamide on days 1 to 3 (PAVEP) for four to six cycles, followed by autologous hematopoietic stem cell transplantation (22). Wens et al. showed that although many different chemotherapy regimens are described in the literature, virtually all patients received a platinum-based regimen, combined with at least another agent (7). In this study, the authors also reported that pelvic recurrence is common in patients after a complete cytoreductive surgery and intensive chemotherapy (7). Radiotherapy has been used in adjuvant or palliative settings, although its benefit remains uncertain (23, 24).

Ovarian cancer during pregnancy presents significant challenges in both clinical counseling and therapeutic planning. As a result, a tailored, patient-specific approach is essential. Non-ionizing imaging modalities (ultrasound and MRI) are preferred for tumor evaluation and can be used in any trimester of pregnancy to evaluate the tumor and its dissemination (25). MRI is considered safe and a valuable diagnostic tool during pregnancy, particularly in oncologic patients. MRI provides excellent soft tissue contrast and allows for the detailed assessment of tumor location, local invasion, and distant spread. Gadolinium-based contrast agents cross the placental barrier and are generally avoided due to potential fetal risks (26). Future research should focus on large-scale prospective trials with detailed data collection to better comprehend the full risks of gadolinium-based contrast agents (27). Surgery is the cornerstone in the management of most gynecological malignancies (9). When indicated, surgery can be safely conducted during pregnancy but carries inherent risks, including spontaneous abortion, preterm delivery, and fetal compromise. Pregnancy-induced physiological alterations in cardiovascular dynamics must be considered when establishing perioperative monitoring protocols (28). In cases of advanced-stage epithelial ovarian carcinoma, pregnancy termination may be advisable if the diagnosis occurs during the first half of gestation (29). Cytoreductive surgery should be deferred until the postpartum period, as intraoperative peritoneal evaluation, extremely high perioperative risks, and complete resection may not be feasible during pregnancy (25). After 14 weeks of gestation, chemotherapy is safe, and there are robust data regarding the use of taxanes, platinum agents, anthracyclines, etoposide, and bleomycin (30). In several studies, the rate of fetal malformations was comparable to that of the general population, demonstrating the relative safety of chemotherapy beyond the first trimester (30, 31).

## 4 Conclusion

SCCOHT is a rare and aggressive malignancy that almost exclusively affects young women and rarely presents during pregnancy. Our case highlights the complexities in managing SCCOHT during pregnancy, illustrating the delicate balance between maternal treatment and fetal safety. The necessity to balance prompt oncologic treatment with considerations for fetal development leads to difficult clinical decisions, compounded by the lack of robust data or standardized treatment guidelines for this scenario. Despite aggressive multi-modality therapy (including cytoreductive surgery, chemotherapy, and immunotherapy), the disease progressed rapidly, culminating in the patient’s death shortly after delivery. Due to the paucity of data, there are no standardized guidelines to help clinicians navigate such an aggressive tumor. However, we acknowledge that the tumor rupture in the first surgery and the delayed start of adjuvant chemotherapy after the cytoreductive surgery may have played a role in the dismal outcome of our patient. Loss of SMARCA4 (BRG1) remains a pivotal diagnostic marker for SCCOHT and offers a molecular target for emerging therapies. Given the current absence of evidence-based protocols, each case must be carefully individualized through a multidisciplinary approach that incorporates gynecologic oncology, medical oncology, maternal–fetal medicine, pathology, and genetics. Further research is urgently needed to better understand the biology of SCCOHT, develop targeted therapies, and establish clinical guidelines that can support decision-making in the context of pregnancy. Prospective registries and collaborative efforts are essential to improve both maternal and fetal outcomes.

## Data Availability

The original contributions presented in the study are included in the article/Supplementary Material. Further inquiries can be directed to the corresponding author.
